# Calcium’s Role and Signaling in Aging Muscle, Cellular Senescence, and Mineral Interactions

**DOI:** 10.3390/ijms242317034

**Published:** 2023-12-01

**Authors:** Kristofer Terrell, Suyun Choi, Sangyong Choi

**Affiliations:** Department of Nutritional Sciences, College of Agriculture, Health, and Natural Resources, University of Connecticut, Storrs, CT 06269, USA

**Keywords:** calcium, aging muscle, senescence, zinc, iron

## Abstract

Calcium research, since its pivotal discovery in the early 1800s through the heating of limestone, has led to the identification of its multi-functional roles. These include its functions as a reducing agent in chemical processes, structural properties in shells and bones, and significant role in cells relating to this review: cellular signaling. Calcium signaling involves the movement of calcium ions within or between cells, which can affect the electrochemical gradients between intra- and extracellular membranes, ligand binding, enzyme activity, and other mechanisms that determine cell fate. Calcium signaling in muscle, as elucidated by the sliding filament model, plays a significant role in muscle contraction. However, as organisms age, alterations occur within muscle tissue. These changes include sarcopenia, loss of neuromuscular junctions, and changes in mineral concentration, all of which have implications for calcium’s role. Additionally, a field of study that has gained recent attention, cellular senescence, is associated with aging and disturbed calcium homeostasis, and is thought to affect sarcopenia progression. Changes seen in calcium upon aging may also be influenced by its crosstalk with other minerals such as iron and zinc. This review investigates the role of calcium signaling in aging muscle and cellular senescence. We also aim to elucidate the interactions among calcium, iron, and zinc across various cells and conditions, ultimately deepening our understanding of calcium signaling in muscle aging.

## 1. Introduction

Muscle tissue offers an interesting yet challenging model for examining calcium signaling, aging, and the associated physiological implications such as fragility. Regarding calcium, unlike a majority of cells, muscle cells are multinucleated and have a specialized endoplasmic reticulum (ER) called the sarcoplasmic reticulum (SR) or smooth ER. With a lack of ribosomes, the SR mainly functions as calcium storage within muscle cells allowing for contractile functions. Throughout aging, in most species, there is a loss of muscle tissue known as sarcopenia. This loss of muscle typically results in increased fragility, risk of injury, and overall mortality risk [1]. Furthermore, chronic low-grade inflammation is associated with aging, known as inflammaging, and is related with decreased time to recover post-injury, decreased strength, and changes in fat deposition/insulin signaling [2,3,4].

Cellular senescence is the stable proliferative arrest of a cell and is also associated with aging and age-related diseases. Within senescent cells, there are many physiological changes including but not limited to metabolic shifts, increased reactive oxygen species (ROS), increased cell size, changes in the fusion/fission of mitochondria, changes in mineral concentration (iron, calcium, zinc, magnesium, and others), an increase in the expression of cell cycle arrest genes such as *CDKN2A*/*CDKN1A*/*TP53*, and an increase in secretions known as the senescence-associated secretory phenotype (SASP) [5,6].

Various factors can induce cellular senescence, typically related to DNA damage, such as telomere shortening through repeated cell divisions or biological agents. Senescent cells are associated with age-related disease, and when removed via senolytic therapy, many have seen benefits such as extended lifespans in model organisms, increased muscle mass, and functional benefits such as speed on treadmill or grip strength [5]. Not all senescent cells are harmful; there is a significant use for cellular arrest during early development and tumorigenesis. Calcium levels, along with other minerals such as iron, have been shown to increase in senescent cells and may play a role in their deleterious effects such as accumulation within the mitochondria, causing dysfunction [6,7].

Calcium signaling has a specialized role in skeletal muscle (SkM) within the organization hierarchy of muscle, moving from large to small: muscle, fascicle, muscle fiber, and myofibril. Within the myofibril are the contractile units of the muscle, the sarcomeres. Thin filaments (actin) are sandwiched between thick filaments (myosin), and during contraction, the two filaments slide across one another. This sliding mechanism is significantly facilitated via calcium signaling. In the mid-20th century, it was discovered that calcium binds to troponin, inducing a conformational change that permits the binding of actin to tropomyosin. This interaction between the two filaments results in muscle contraction [8]. Calcium levels must be controlled for intensity of contraction, duration, and relaxation.

Indeed, calcium does not function in isolation; it is part of intricate mineral–mineral interactions that have been increasingly elucidated in recent years, providing insights into the complex interplay mechanisms involved. This review highlights two additional minerals, iron and zinc, due to their biological significance, current research relevance, and public health implications; calcium, iron, and zinc are among the top-selling minerals for supplementation [9,10]. Electrolytes such as potassium, magnesium, and sodium are not the focus of this review, which instead focuses on minor elements, specifically iron and zinc, as they are present in the highest concentrations throughout the aging muscle [11]. Both iron and zinc have intricate relationships with calcium, calcium channels, aging, and cellular senescence. Many of these channels, originally thought to transport only a single mineral, are now understood to interact with multiple cations and exert influence over each other. This underscores the complexity of mineral interactions and their critical roles in biological processes.

## 2. Calcium Signaling and Aging Muscle

### 2.1. Role of Calcium in Muscle

The movement and buffering of calcium are paramount for the upkeep of cellular homeostasis. Calcium transporters, channels, exchangers, pumps, and binding and buffering proteins are all regulated to control the flow of calcium. As a main control system for the calcium release of internal stores in SkM, the SR can be classified into two types according to localization within the sarcomere. The longitudinal SR, located around contractile units, plays a role in contraction and relaxation, while the junctional SR, located in tight proximity to sarcolemma invaginations known as transverse tubules, regulates contraction initiation [12]. The SR in smooth muscle cells is more akin to a typical cell’s ER as these cells lack myofibril-forming sarcomeres and thus are arranged differently. The nuclear envelope of cardiac muscle is also interconnected with the ER/SR, acting as a calcium storage system [13]. Striated muscles (those containing regular arrangements of actomyosin fibers), including cardiac and skeletal muscles, contract as a whole via voltage- and calcium-dependent excitation–contraction coupling. In contrast, smooth muscle contraction is more sustained and slower due to differences in the contractile system.

The release of calcium requires both tight regulation, as well as reception via various calcium-sensitive proteins. The amount of calcium released, duration, microenvironment, and oscillation all take part in calcium signaling [14]. Calcium release from the SR can occur via ryanodine receptors (RyR) or inositol-1,4,5- triphosphate receptor (IP3R). RyR1 and RyR2 isoforms are particularly important for excitation–contraction coupling in skeletal and cardiac muscles. An action potential traveling through transverse tubules results in SR release by mechanically coupling to dihydropyridine receptor (DHPR) in SkM [15]. IP3R-mediated SR calcium release is activated via inositol-1,4,5- triphosphate (IP3). Other ligands such as cytoplasmic calcium can also cause IP3R calcium release, where, depending on the concentration of the IP3, IP3R can cause various intensities of calcium signaling [16].

Upon calcium accumulation in the SR, buffer proteins like calsequestrin or calreticulin, which have lower binding affinity but high capacity for calcium, serve a storage role. High-binding affinity proteins such as parvalbumin and S100G function as transducers of the calcium signal and are often localized in the cytoplasm [17]. Calcium sensors such as calmodulin (CaM), troponin C, and neuronal calcium sensor (NCS) undergo conformational changes when bound with calcium, triggering their individual functions such as aiding in neurotransmitter release, gene regulation, and muscle contraction [18,19]. This comprehensive understanding of calcium signaling players helps elucidate its importance in maintaining cellular homeostasis and physiological processes in muscles.

The critical role of calcium signaling in muscle contraction has been well studied. The metal ions in SkM function to aid in contraction within the sliding filament model. Briefly, the contraction is generated by the sliding of actin-containing thin filaments and myosin-containing thick filaments. Myosin motors transiently interact with the actin filaments triggered by calcium-regulatory structural changes. Following electrical stimulation, calcium ions are released from intracellular stores and bind to the head of troponin, changing structurally and allowing the availability of myosin binding sites on actin.

Recently, Brunello and colleagues advanced the understanding regarding activation of SkM [20]. To investigate the steady-state calcium dependence of regulatory structural changes in thin filaments, they used probes on the C or E helix of troponin C, which are mainly sensitive to the opening of the lobe due to the calcium binding site. They propose that thin filaments are only partially activated by calcium and that the full activation and cooperation require the binding of force-generating myosin motors. This leads to a dual-filament model of contraction activation in SkM in which the steady force is controlled by two positive feedback loops in thin and thick muscle triggered by calcium. During contraction, calcium ions partially activate the thin filament, and the force generated by the actin-bound motors triggers the release of folded myosin motors in the thick filament, increasing the fraction of actin-bound motors and initiating a positive mechano-sensing feedback loop. This allows for more myosin motors to bind to actin, triggering a positive myosin-sensing feedback loop, all to aid in an increase in the generation of force.

Specific to the structural changes in thin filaments upon calcium binding, electron cryomicroscopy has allowed a deeper understanding [21]. In cardiac muscle, troponin, which consists of three subunits—(1) troponin C, calcium binding subunit; (2) troponin I, inhibitory subunit; and (3) troponin T, tropomyosin binding subunit—plays a crucial role. When calcium binds to the N lobe of troponin C, it dissociates, from the C-terminal, one third of troponin I (tNiC) due to the binding of a short N-terminal portion of tNiC to the N-lobe of troponin C. This causes tropomyosin to move around on the actin filament surface together with the N-terminal chain of troponin T near the head-to-tail junction of tropomyosin, thereby exposing some of the myosin head binding sites and facilitating actin–myosin interactions. The shift distance in a calcium-bound environment compared to a calcium-free environment seems to vary depending on the position along the coil, with the azimuthal shift around the head-to-tail junctions being smaller than parts near the troponin core. Similar shift distances have been shown in previous studies, likely due to troponin T binding to tropomyosin in this region [22]. Its N-terminal side also binds to the actin filament, thus restricting the tropomyosin shift. In calcium-free states, troponin keeps tropomyosin in position, fully blocking the access of the myosin head to the actin filament; upon the binding of calcium, tropomyosin shifts to a position that allows myosin head access but is not a fully open position to allow the binding of the myosin head.

The key role of intracellular calcium homeostasis in myofibril organization was suggested in the obscurin knock-out (KO) mice model, a model of SR dysfunciton [23]. Obscurin is a sarcomeric protein with localization patterns distributed among the M band and minorly at the Z-band. In the murine null model, an age-dependent reduction in endurance was observed when compared to the wildtype (WT) during exhaustive exercise [24]. It has been found that not only does the structural function of sarcomere assembly play a significant role in fatigue, but intracellular calcium levels are also affected. The obscurin KO led to an increased intracellular calcium level, and the average changes in the amplitude of calcium via electrical stimulation were lower in the KO mice compared to the WT mice. Additionally, a decrease in the expression of the sarcoplasmic/endoplasmic reticulum Ca^2+^- ATPase (SERCA) protein was observed in the KO mice, suggesting that the SR dysfunction led to calcium dyshomeostasis, which in turn had a detrimental impact on muscle endurance. 

With regard to insulin resistance, SkM is important for glucose uptake caused by insulin signaling. This process involves the phosphorylation of insulin receptor substrate (IRS1) by insulin receptor (IR)-activating phosphoinositide 3-kinase (PI3K), forming phosphatidylinositol-3,4,5-triphosphate (PIP3), activating protein kinase Akt, and leading to the translocation of GLUT4 onto the cellular membrane, which increases glucose influx. Calcium regulation appears to play a role in insulin stimuli [25].

Calcium may also have a role in SkM growth. Insulin-growth like factor 1 (IGF1) recruits the PI3K-mammalian target of rapamycin (mTOR) signaling axis to implement muscle hypertrophy or growth [26]. The potential role of calcium in this pathway is within the calcium–calcineurin relationship, although there is still no consensus on this [27]. A more well-documented pathway is that of IGF-1, which causes IP3R calcium signal upstream of gene expression mediated by NFAT, supporting the relation between calcium and muscle differentiation and growth [28]. In the next section, we will discuss muscle aging and SkM.

### 2.2. Aging’s Effect on Muscle

One tissue within a whole organism, aging muscle, experiences similar deleterious changes with age akin to other tissues, and these changes are summarized in Table 1. Age-related cellular events such as DNA damage, genomic instability, loss of proteostasis, and mitochondrial dysfunction, among others, are thought to be the initiators of age-related damage [29]. Aged muscle tissue is also unique due to its post-mitotic nature and inability to divide. As dividing cells are prone to telomere shortening, one might assume that the quiescent myofibril telomere would be protected from shortening. Still, telomere shortening is seen In skeletal tissue of adults, likely due to an increase of free radicals with aging [30]. Instead of mitosis, muscle size increases through the fusion of myoblasts. As muscle size increases, such as in growth from weightlifting, myoblast recruitment and an increase in the size and number of contractile myofibril cause the growth [31]. The age-related loss of muscle mass, strength, and function is termed sarcopenia, and since the inception of this idea, its definition has been consistently altered [32].

**Figure 1:**
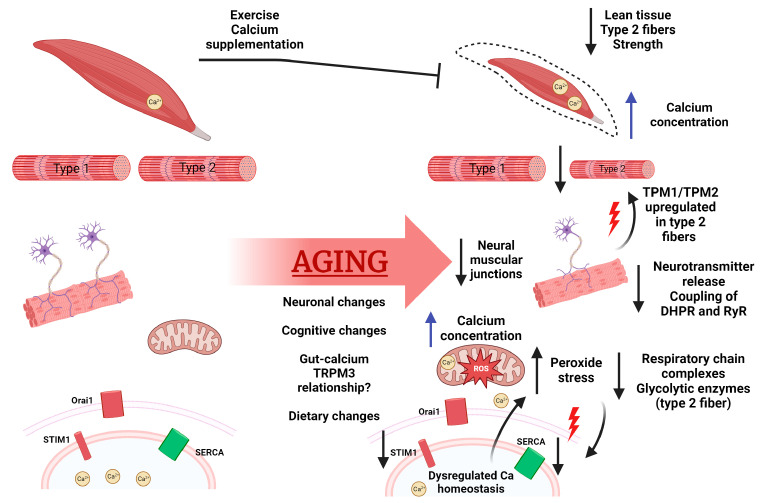
Calcium homeostasis and function in aging. With age, we see decreased lean tissue, known as sarcopenia, specifically loss of type 2 fast-twitch fibers. Calcium concentration has been seen to increase in aged muscle, with age-related denervation resulting in upregulated Tropomyosin 1/2 and Troponin C1/C2. Furthermore, denervation leads to decreased coupling on DHPR and RyR. An accumulation of mitochondrial dysfunction is also seen, with changes in calcium content, increased ROS, and changes in respiratory functions. This mitochondrial damage has been seen to cause declines in SERCA protein levels. Decreased SERCA levels and age-related loss of STIM1 promote dysregulated calcium homeostasis, leading to further cellular stress such as mitochondrial damage, DNA damage, and apoptosis/atrophy. In addition to musculature, there are whole-body changes with respect to calcium such as neuronal changes in calcium content with links to cognition decline, age-related calcium dysregulation correlated to neurodegenerative diseases, gut–mineral relationships, and changes in calcium uptake overall. Figure created with BioRender.com.

Sarcopenia patients often experience mobility issues, an increased risk of falls and fractures, and impaired ability to perform daily physical tasks such as moving from sitting to standing. Sarcopenia frequently co-occurs with other age-related ailments, including increased body fat, insulin resistance, immune system issues, and cancer, to name a few [29,30]. Furthermore, changes in diet composition, eating patterns, lifestyle, and exercise habits occur with age, affecting SkM [43]. A loss of fast-twitch muscle (type 2) and increased fat deposition are also found with the aging muscle. Proteomic differences between young and aged individuals within the two fiber types reveal that slow fibers (type 1) increase the expression of protein homeostasis factors and carbohydrate metabolism as compared to fast-twitch fibers within aged populations [34]. In both tissue types, similar decreases in oxidative phosphorylation and mitochondrial complex protein due to aging have been observed compared to young individuals. Neuromuscular junctions and motor units also decrease with age [44]. These age-related changes in skeletal muscle are reviewed in Figure 1.

Exercise is well characterized for the treatment and prevention of muscle loss. Not only is muscle loss common with aging, but so is bone density loss, or osteopenia. The term “osteosarcopenia” has been coined to reflect the close relationship between these two conditions [45]. In elderly community-dwelling males with sarcopenia, exercising 2–3 times a week has been shown to increase both bone mineral density and SkM mass [46]. The use of vitamin D and calcium supplementation has been found to improve bone and muscle health in both men and women, although not as substantially as exercise, suggesting that the aging of muscle is not necessarily a result of a deficiency in these specific nutrients in the aging population [47].

Intracellularly, there is an increased susceptibility to oxidative stress, impaired mitochondrial function, and protein modifications. At the organism level, there is inflammation, immunosenescence, an increased number of senescent cells, and changes in anabolic hormone levels. These factors, ranging from molecular to tissue to organism activity and comorbidity issues, can lead to the muscle atrophy seen in aging populations [48].

### 2.3. Aging’s Effect on Calcium

As an action potential travels down a presynaptic neuron, it triggers the release of neurotransmitters and calcium into the synaptic cleft. These neurotransmitters then move into the muscle cell, travel down T-tubules, and interact with DHPR, RyR, and IP3R to aid in calcium release and muscle contraction. Throughout these events, the sarcolemma becomes depolarized, and the action potential from the neuron moves through to the muscle. With age, there are decreased calcium release, neurotransmitter release, synaptic vesicle function, and an uncoupling of RyRs to DHRPs [44]. Interestingly, DHPR-null mice, which lack calcium influx, showed no alteration in excitation–contraction coupling (with intact transient receptor potential canonical (TRPC) channels, Orai channels, and SERCA channels) and no effect on SR filling or physical tests in both young and aged mice. Furthermore, no compensatory regulation or increased protein expression of other excitation–contraction coupling channels was observed [49]. In a loss of Schwann cell phenotype, present in CRD-Nrg1 KO mice, which lack functional contraction mechanisms, a rescued contraction was found via dual loss of both Schwann cells and DHPR (CRD-Nrg1−/− Cacnb1−/− genotype) [50]. As mentioned above, voltage-gated RyR calcium release is not the only method of SR calcium release; IP3R can also facilitate this process. Limited studies have been conducted to elucidate the relationship between IP3R and aging within the SkM. However, IP3R has been linked with deleterious effects within cellular senescence and longevity, both due to the calcium regulation within the mitochondria, which will be discussed later in this review [16].

Store-operated calcium entry (SOCE) is a mechanism that functions to increase calcium uptake following calcium release from ER/SR. The two key players in SOCE are STIM1, a calcium sensor located in the ER/SR, and Orai1, a calcium conducting channel. Comparing aged mice to young (25 months vs. 2–3 months), protein levels of STIM1 were found to be decreased in extensor digitorum longus, while Orai1 levels remained unchanged [51]. Despite the decreased STIM1 expression, the function of SOCE, as measured via calcium imaging, showed no change in basic properties. However, another group looking at a different muscle, the flexor digitorum brevis, in a similar age group (young: 2–4 months or aged: 26–27 months) reported slightly different results [52]. Initial responses to osmotic shock resulted in similar spark signaling, but subsequent calcium spikes in aged muscle were blunted or not observed. Resting calcium levels were not different between the young and aged mice but calcium store, measured via caffeine/ryanodine mobilized SR Ca^2+^, was seen to be decreased in aged muscle [52]. The discrepancy in calcium flux observed between these two studies may have been due to differences in isolation methods (mechanical vs. enzymatical) or perhaps due to differences in the muscles studied. SOCE is responsible for more than just maintaining calcium concentrations, as evidenced by changes in the immune system and muscular structure in the relevant KO models [53,54]. Additionally, decreased expression of mitsugumin 29 (MG29), a synaptophysin-related membrane protein that interacts with Bin-1 to maintain T-Tubule structure, was found in aged SkM [52]. T-tubules function to maintain the proximity of the sarcolemma and SR, allowing for efficient calcium release throughout the whole muscle cell [55].

Understanding the many calcium-related dysregulations associated with aging, one may wonder how calcium concentration changes. Recently, it was shown that muscle fiber concentrations of calcium increase throughout the lifespan of mice [56]. In contrast, in whole muscle (quadriceps) collected from male C57BL/6J mice fed a standard chow diet ad libitum, calcium levels were not significantly affected by age [11]. However, in flexor digitorum brevis fibers isolated from young (3 months), middle (12 months), or aged (24 months) C57BL/6J mice, increased concentrations of Ca^2+^ were seen, from 121 nM in young cells to 409 nM in aged cells [6]. The lack of significant change in whole muscle tissue could come from the wide array of cell types inside muscle tissue versus isolated fibers in the latter study. Studies directly looking at muscular calcium levels in humans are required. However, in a longitudinal study looking at serum calcium and muscle loss, it was found that regardless of sex, individuals with the lowest calcium levels had more significant muscle loss than those with high calcium levels [57]. More research is needed before a direct line causality can be made; however, the correlations between calcium, muscle contractility, muscle reduction, and aging are seen across multiple organisms.

As aging is associated with increased inflammation, ROS, and the previously mentioned calcium dysregulation, the direct relationship between ROS and calcium SR flux was investigated. To study the relationship involving ROS and/or SR calcium flux, flufenamic acid was also used in an aged mouse model [58]. Flufenamic acid is an anti-inflammatory reagent with anti-prostaglandin synthesis properties and a modulator of TRP channels such as TRPC, TRPM, TRPA, etc. The treatment of flufenamic acid caused decreases in muscle calcium concentration in adult and aged time points— 12 and 24 months, respectively—as compared to age-matched control. There was also an increase in inflammatory markers, such as IL-6 and TNF-α, in aging plasma. Increased ROS levels also correlate with increased age regardless of treatment. However, when comparing adult mice to young mice, there was a rescue effect (lowering sodium/ROS/calcium) of flufenamic acid that was only seen in the young group, where the aged were unable to be rescued. The use of flufenamic acid and its rescue in mid-age point us in the direction of these TRP channels and their relationship with the pathogenesis of the aging phenotype. However, the anti-inflammatory effects of flufenamic acid likely also play a role as it has been used in inflammation-associated musculoskeletal and joint disorders [6,58]. More research is needed to elucidate specific relationships TRP channels may have with aging.

## 3. Cellular Senescence

### 3.1. Aging Muscle and Senescence

Cellular senescence was observed in the mid-1900s when human fibroblasts ceased to proliferate after several passages [59]. This replication-induced senescence occurred due to telomere shortening, but other stimuli such as oncogene activation, oxidative stress, DNA damage, or other stressors can induce cellular senescence. Senescence is typically induced by a complex network of factors in which cell cycle regulators, including p53, p16, and p21, inhibit cyclin-dependent kinase complex formation, thereby arresting proliferation. The phenotype of senescent cells is heterogeneous and can include altered mitochondrial metabolism, cell morphological changes, reactive oxygen species production, a secretory phenotype, and altered chromatin and gene expression [7]. In aging SkM, trends such as a decrease in muscle mass, altered insulin signaling, and inflammation are observed. While these age-associated issues are common in a majority of animals, the correlation with cellular senescence is still not fully understood.

Some studies on aged SkM have reported increased senescent markers, such as upregulated gene and protein expressions of p16, p21, or p53 in various organisms, while others have shown no detection of p16 or SA-β-gal [60,61,62,63]. The nuclei of aged humans and mice SkM were examined for telomere dysfunction and found to have a higher proportion of nuclei positive for the co-localization of γ-H2AX and telomeres, indicating damage. Although no SA-β-gal was found, there was a loss of nuclear HMGBI and Lamin BI and an increased centromere length in old SkM, suggesting that SkM cells are prone to senescence with advancing age [60]. Moreover, the removal of senescent cells has been seen to counter the age-associated increase in senescence-related genes. Such senolytic therapy did increase grip strength but did not alter age-related reductions in SkM mass and myofiber size [5,60]. These findings highlight the complex nature of cellular senescence and its impact on aging.

Following a muscle injury induced by muscular injection of cardiotoxin in both young and old mice, senescent cells were observed to appear after 3 days and stay elevated until 7 days post-injection, after which they decreased [2]. In the aged muscle, senescent cells were more abundant and persisted longer in the injured muscle. The telomere damage response was greater in regenerating muscle in old mice, as opposed to young. To understand the role of senescent cells, young and old p16-3MR mice, a model that allows visualization and elimination of senescent cells, were treated with ganciclovir (GCV) to reduce the presence of senescent cells [64]. This treatment rescued defective muscle regeneration, reduced inflammation and fibrosis, and enhanced force generation in old mice [2]. It also accelerated the regenerative ability in young mice, which implies that senescent cells have detrimental effects in both aged and young mice [2]. Acute damage may have a different role from chronic muscle damage; in chronic muscle damage, which may be induced naturally via chronic inflammation, increased SA- β-gal activity and p-16 were observed in a chronic damage model of micro punctures [65]. Senolytic treatment led to increased size in regenerating myofibers and decreased inflammation. This finding led researchers to conclude that the interruption of senescent cells in muscle niche, for both mild as well as chronic injury, is beneficial, challenging the classical idea that senescent cells are beneficial when transiently present after acute injury.

The two main regulated traits of senescent cells across age and time points, based on pathway enrichment analyses, serve two functions: inflammation and matrix remodeling/fibrosis [2]. SASP affects muscle regeneration likely via inflammation interactions in muscle stem cells, inducing proliferative arrest via DNA damage. In GCV-treated stem cells, these cells had higher proliferation ability ex vivo compared to stem cells from vehicle-treated mice. Moiseeva et al. thus suggested that senescent cells restrain muscle regeneration through paracrine pro-inflammatory and pro-fibrotic SASP functions that blunt stem cell proliferation.

Increased intracellular calcium concentration has been observed in the cytosol and mitochondria of senescent cells. In senescent human mammary epithelial cells, calbindin 1 calcium-binding protein (buffering cytosolic calcium) was seen to be upregulated [66]. The rise of intracellular calcium concentrations can be triggered via influx through plasma membrane or by intracellular calcium stock from the ER. Recently, there has been evidence showing which channels are causing rises in calcium in senescence. From the ER, ITPR1-3 has been seen to release calcium during cellular senescence, and the knockdown of this protein allows escape from oncogene-induced senescence and a delay in replicative senescence in fibroblasts [67]. This release is linked with the mitochondrial increase in calcium via ER–mitochondria contact.

### 3.2. Mitochondria Dysfunction and Senescence

Mitochondria store calcium ions; however, this is not an inert relationship. Instead, calcium is understood to play roles in ATP production within the mitochondria and undergo dynamic flux [68]. Thus, within senescent cells, where there are typically dysfunctional mitochondria and increased calcium concentration, perhaps there is an interplay between the two. Excess calcium can trigger apoptosis via the opening of the mitochondrial permeability transition pore (mPTP), allowing for the bulk efflux of particles, including protons and calcium, from the mitochondrial matrix [69]. Cellular senescence is characterized by the dysregulation of calcium flux, changes in mitochondrial membrane polarization, increased production of ROS, and a resistance to apoptosis mechanisms, including the one just described.

Certain stressors, such as increased ROS, mitochondrial fission, or the fragmentation/generation of smaller mitochondria from larger precursors, can occur. These processes typically serve cellular replication purposes but also play roles in mitophagy. Mitochondrial fusion is a process that generates larger mitochondria from smaller ones, which is associated with increased Krebs cycle activity and ATP production. Fusion may alleviate mitochondrial injury by recombining injured mitochondria with healthy components [70]. During cellular senescence, these processes are altered, with the mitochondria of senescent cells typically increasing in size, volume, and number of dysfunctional mitochondria [71].

As calcium concentration can interact with mitochondrial function, how mitochondria regulate calcium flux is important. Under normal circumstances, the import of calcium into the mitochondria is seen to be mainly through the mitochondrial calcium uniporter (MCU), but also there is support for transporters such as LETM1 (leucine zipper-EF-hand-containing transmembrane protein 1) acting as calcium/hydrogen antiporters mediating mitochondria calcium influx [72]. Mitochondrial sodium/calcium exchanger normally functions to remove calcium from mitochondria but has been suggested to work in the opposite direction when the mitochondria are depolarized [73]. However, from MCU-KO studies, there is a lack of rapid uptake of calcium in the absence of MCU, suggesting that even with the other potential mitochondrial calcium influx proteins, MCU is the more significant pathway [74]. In cell KO models of IP3R or MCU, the cells are still viable, but show decreased growth and oxygen consumption rates, indicating there are other sufficient means by which calcium can enter the mitochondria [75].

The inositol 1,4,5-triphosphate receptor type 2 (ITPR2) calcium channel is found on the ER and SR of cells and functions to promote mitochondria contact and calcium transfer. Studies on ITPR2 KO mice by Ziegler et al. showed an increased lifespan in female (but not male) mice compared to WT mice, while WT males live longer than WT females, suggesting that ITPR2 could still contribute to lifespan differences between male and female mice [67]. These KO mice also exhibited reduced cellular senescence and reduced mitochondria–cytoplasmic reticulum contacts. Conversely, increasing these contacts via a synthetic linker increased premature senescence. Contacts between ER and mitochondria aid in triggering senescence involving mitochondrial ROS/p53 and partially NF-kB-dependent SASP. This result implies a potential pathway by which mitochondrial calcium changes leads to energetic changes, and more ROS production that may contribute to cellular senescence [67].

Eukaryotic cells have evolved several mechanisms for handling excess ROS. One such is via superoxide dismutases (SODs). SOD1 contains copper and zinc subunits that are responsible for catalyzing the disproportionation reaction, and it is localized on the mitochondrial matrix, while SOD3 is in the extracellular space [76]. Yamamoto-Imoto et al. showed that a specific transcription factor, MondoA, is important for the prevention of cellular senescence [77]. MondoA expression decreases with age, affecting PRDX3, a part of the enzymatic antioxidant family, which is involved in redox signaling and cell-cycle progression [77]. PRDX3 is localized on the mitochondria, and when MondoA was suppressed via siRNA, mitochondrial function was impaired. Changes in mitochondrial physiology, respiration, interactions with other organelles, and genetic factors all occur during senescence. With age comes a disrupted defense against oxidative stress, and at the same time, increased production of ROS; this leads to increased stress on the cells promoting cellular damage and dysfunction. 

Linking mitochondria, senescence, and the aging muscle, Debattisti et al. showed that the dysregulation of mitochondrial Ca^2+^ uptake, via a mitochondrial calcium uptake 1 KO mouse model, decreases myofiber contractility and is related with muscle loss [78]. Mitochondrial-calcium-uptake-1-deficient patients have also been seen to experience muscle weakness and dysfunction [79]. Based on the relationships between senescence and mitochondrial dysfunction, mitochondrial dysfunction and muscle dystrophy, a link between mitochondrial calcium and the aging muscle phenotype may be supported, wherein the progression of biological age causes senescence, low-grade inflammation, and mitochondrial dysfunction. including calcium dysregulation, that aids in the progression of muscle tissue dysfunction, dysregulation, and sarcopenia.

## 4. Crosstalk between Minerals

We have discussed the role of calcium signaling in aging muscle and senescent cells and reviewed the interrelationship. Given that various factors regulating calcium signaling can be impacted by other minerals in the cell, such as zinc and iron, it is beneficial to understand how these other minerals play a role in aging muscle and whether these roles are mediated by intracellular calcium flow or calcium signaling-associated proteins.

Zinc plays multiple roles, including structural, enzymatic, and cellular signaling. Zinc deficiency is more common in the elderly and has implications with immune system function [80]. While muscle levels of zinc are mostly maintained during whole-body zinc deficiency, zinc deficiency may work to lower the activity of metalloenzymes such as lactic dehydrogenase [81]. In contrast, the activity of mitochondrial glutamic dehydrogenase remains unaltered during deficiency, suggesting that zinc deficiency does not affect all proteins equally; more work is needed to understand the role of zinc deficiency and its associated protein function within muscle [82]. Interestingly, in elderly community dwellers, zinc supplementation reduces multidimensional fatigue inventory (MFI) scores [83]. The MFI assesses levels of general, physical, and mental fatigue, as well as physical activity and motivation, based on a five-point Likert-scale-based 20-question test. This suggests that, specifically in an aged population where deficiency may be more prevalent, zinc supplementation may be protective against the deleterious cross-organ-system effect of deficiency.

Within senescent cells, zinc concentration has been observed to increase as much as five to eight-fold depending on the cell line, from 0.176 μg per 5 million in young cells to 0.725 μg in aged fibroblasts, which may be critical for senescence progression and phenotypes [84]. Aligning with this, zinc treatment in human bronchial epithelial cells was seen to induce cell cycle arrest with high p53/p21 expression [85]. Zinc is also understood to increase in the mitochondria of senescent cells, leading to increased ROS production. Zinc treatment induces NADPH oxidase activity and NF-kB activation, promoting inflammation [86]. Great details about zinc homeostasis and mitochondria function are covered in a review by Dabravolski et al., where the consensus is that the dysregulation of zinc concentration leads to mitochondrial damage and an accelerated senescence phenotype [87].

Iron serves an essential role in the body due to its relationship with heme and its oxygen-carrying capacity. However, iron’s chemical properties make it prone to react and produce hydroxyl free radicals through the Fenton reaction. When unregulated, this can cause DNA and protein damage, as well as lipid peroxidation leading to cellular responses like apoptosis or senescence. Iron deficiency is one of the most frequent nutritional deficiencies worldwide and the second most common cause of anemia in the aged population, and still, iron levels in SkM have been seen to increase with age [88,89]. This iron overload increases ferroptosis, an iron-induced cell death pathway, and seems to impair muscle stem cell function [90]. Specifically, iron accumulation triggers p53-SLC7A11-mediated ferroptosis in muscle [91].

Iron regulation occurs at multiple levels, from absorption and storage to utilization in various organ systems. The primary iron-regulatory hormone is hepcidin. Hepcidin is regulated by cellular iron, circulating transferrin-bound iron, and inflammation. Increased hepcidin reduces intestinal ferroportin, thereby reducing iron release from enterocytes into the bloodstream. Inflammation increases hepcidin concentration, leading to altered iron absorption [92]. While senescent cells can produce pro-inflammatory secretions, the relationship between these secretions and hepcidin is not well studied.

Within senescent cells, iron levels have been seen to increase in various cellular models, with some showing 20- to even 50-fold increases in iron concentration [84,93]. Impaired ferritinophagy and the inhibition of ferroptosis are also reported and thought to be due to senescence-associated lysosomal dysfunction. This buildup of iron in senescent cells is also understood to increase mitochondrial iron concentrations, causing further damage and a building up of cellular damage supporting senescence progression [94].

In addition to the direct impact of these metals on aging muscle and senescent cells, the following subsections will further introduce the crosstalk between the two metal ions and calcium signaling in muscle or other tissue-originated cells so as to better understand their roles in aging muscle. These potential crosstalk interactions may be important in aging and cellular senescence as the concentrations of various minerals are dysregulated and may interact with calcium in deleterious ways as presented in the studies below.

### 4.1. Zinc

#### 4.1.1. Interaction with Calcium Channels and Calcium-Binding/Dependent Proteins

The movement of zinc ions (Zn^2+^) across cellular membranes is facilitated by zinc transporters [95]. However, it has been identified that zinc can also be transported through calcium channels [96]. The permeability of zinc through the voltage-gated calcium channel (VGCC) was suggested by the observation of the voltage-dependent blockage of Zn^2+^ in mouse myotubes and rat primary cortical cultures [97]. This entry of zinc through VGCC was shown to occur in the presence of calcium. A specific subtype of VGCC, L-type calcium channel (LTCC), was reported early on to be shared by both Ca^2+^ and Zn^2+^ [98]. In a myriad of models, LTCC was seen to cause an influx of Zn^2+^ [98,99]. More recently, LTCC agonists have been employed and have been seen to prevent Zn^2+^ waves, supporting earlier research connecting the two [100].

Transient receptor potential proteins (TRP) are a class of 28 cation-permeable membrane channels and are another example of non-selective cation channels [96]. For example, TRPM6 and TRPM7 primarily transport magnesium, but also mediate calcium and zinc transduction [101,102,103]. TRPM2, primarily activated by nucleotides such as adenosine diphosphate ribose, oxidative stress, or others in the presence of calcium, contains the conserved Zn^2+^-binding domain that is essential for structural integrity and channel activity [104]. TRPM2 on the membrane of lysosomes can be potentiated by elevated Ca^2+^ inducing Zn^2+^ release to a toxic level progressing towards apoptosis [105].

SOCE, described previously, functions to refill the calcium stores. In human salivary cells and rat submandibular glands, SOCE channels were proposed to be inhibited by Zn^2+^ [106]. Zn^2+^ acted as a competitive inhibitor of Ca^2+^ influx without permeating the channels, suggesting the interaction of Zn^2+^ with extracellular sites of channels. More specifically, the inhibitory effects of Zn^2+^ on Orai1 have been revealed in human esophageal carcinoma cells [107]. Fluorescence imaging shows rapid inhibitory effects of extracellular Zn^2+^ on Orai1-mediated SOCE accompanying intracellular Ca^2+^ oscillation through zinc binding with specific regions of outer Orai1, suggesting that zinc can inhibit cancer cell proliferation by preventing hyperactive Ca^2+^ signaling.

Previously, cations including zinc and cadmium were suggested as potentiators for skeletal muscle twitching in isolated rabbit skeletal muscle cells [108]. It was examined that shifts in cations from calcium and magnesium to zinc and cadmium occurred with higher binding capacities to the SR, inducing the prolonged activation of muscle contraction. In the same manner, an in vivo study in rats showed a significant switch from calcium to zinc contents specifically in SR, implying an antagonistic effect of zinc on calcium in skeletal muscle [109]. The antagonistic potential of zinc was also observed in smooth muscle [110]. Zinc ions prolonged caffeine-induced contraction of muscles in guinea pigs by blocking the release of calcium from stores. Based on this finding, cardiac RyR2, which functions to release calcium from the SR into cytosol, was suggested as a target [111]. Indeed, high concentrations of zinc decreased ryanodine binding on RyR2 in cardiac muscle [112]. The idea that zinc regulates RyR2-mediated calcium release also proposes that RyR2 has both high-affinity and low-affinity zinc binding inhibition sites, modulating calcium homeostasis in cardiac contraction [113].

RyR2 is not the only SR Ca^2+^-permeable channel regulated by Zn^2+^. An increase in MG23 expression and activity has been observed when cytosolic Zn^2+^ levels rise [114]. This finding is supported by the presence of a common HxxE amino acid sequence conserved on Zrt-/Irt-like protein (ZIP) 1, 2, and 3, known as zinc transporters, where the Zn^2+^ binding sites are located [115]. MG23, along with RyR2, adds more explanation on the diastolic SR Ca^2+^ leakage. This inhibition by zinc may be important as zinc concentrations increase in senescence and some aging models.

Calreticulin is a Ca^2+^-binding protein in SR/ER that contributes to calcium homeostasis along with calsequestrin and histidine-rich Ca^2+^-binding protein (HRC). Zn^2+^ binding to calreticulin has been suggested, and its responsive regions have been also validated. Structural stabilization through the multimerization of calreticulin has been shown to be attributed to Zn^2+^ binding [116,117,118]. Zn^2+^ also binds to calsequestrin, but its functional modification has not yet been elucidated [119]. HRC possesses Zn^2+^-binding sites that are different from Ca^2+^, with lower affinity [120].

CaM, a primary Ca^2+^-binding messenger protein, has other cations suggested as potential allosteric regulators, including Zn^2+^ binding to its EF-hand motif [121,122]. The inhibition of CaM by zinc was first proposed in a study of erythrocytes, which resulted in reduced Ca-ATPase activity [123]. The reduction in CaM and cAMP levels upon the presence of excess zinc was later examined in mice [124,125]. Due to the inhibition of CaM by zinc, the following pathway of CaMKII modulation has been studied, and CaM-independent CaMKII regulation by zinc has also been illuminated [126]. Upon higher concentration of zinc, the activity of CaMKII biased toward autophosphorylation, with altered mobility on the α subunit.

Another primary Ca^2+^-binding messenger, S100 family protein, which includes calprotectin and calcyclin, has also been recognized as a Zn^2+^-binding protein. This is because its affinity for zinc is even higher than that of CaM [127,128]. The responsible crystal structure of the Zn^2+^-EF-hand was validated in various subtypes of S100 proteins [129,130,131]. This evidence supports the potential regulation, by zinc, of S100-mediated proteins such as TRTK-12, p53, mitochondrial ATAD3A, and ribosomal S6 kinase, as well as the roles of zinc in various S100-related pathologies [128].

In neurons, upon calcium flux generated by VGCC and metabotropic receptors, specific NCS responds to different localizations, ranges, and durations of the calcium elevation. NCS regulates signaling targets with diverse physiological outcomes of particular calcium signals. It has been hypothesized that NCS can also sense the fluctuation of intracellular zinc, which diversifies its function [132]. The dimerization of NCS-1 in neurons and retinal photoreceptors is increased by high concentrations of zinc but not by calcium elevation under oxidative conditions, functioning as a redox-regulatory protein [133,134]. Recoverin, a modulator of Ca^2+^-sensitive rhodopsin deactivation, was proposed to be affected by zinc, as Zn^2+^ specifically interacted with recombinant recoverin, inducing lowered stability of this protein [135]. The interaction of zinc with various calcium-binding proteins may have significant implications for a range of physiological and pathological processes related to calcium homeostasis.

#### 4.1.2. Zinc Transporters and Calcium

Not only zinc itself, but zinc transporters can also participate in crosstalk with calcium signaling. Some ZIP transporters may interact with CaMKII. In myocardial ischemia/reperfusion injury cellular models, zinc deficiency induced ER stress leading to ER Ca^2+^ release through enhanced RyR [120]. Activated CaMKII upon increased Ca^2+^ levels further stimulated STAT3, a transcription factor increasing ZIP9 expression. ZIP9 comprised a feedback loop to allow more Zn^2+^ uptake, adapting to the zinc-deficient environment. The same study group found that Ca^2+^ mobilization triggers a reduction in ZIP13 protein expression, and this ZIP13 reduction activates CaMKII, contributing to ischemia/reperfusion injury [136].

Zinc Transporter (ZnT) families interact with cellular Ca^2+^ in different ways. It was addressed that LTCC-mediated Zn^2+^ transport is under the regulation of ZnT1 [137]. A potential mechanism has also been proposed wherein ZnT1 interacts with the β-subunit of LTCC [138]. The sequestration of the β-subunit reduces its capacity to chaperone the α1-subunit to the plasma membrane. This decreases the surface expression of LTCC. Newer findings suggested that ZnT1 can also exchange zinc and calcium directly [139].

### 4.2. Iron

#### 4.2.1. Iron’s Effect on Calcium Signaling and Transport

In the view of public health, the interaction between calcium and iron has been discussed with their inhibitory effects on the absorption of each other. The concern of inhibited dietary iron absorption by calcium is related to intestinal divalent metal transporter 1 (DMT1), a main transporter involved in dietary iron uptake [140,141,142]. It was shown that calcium’s inhibition of DMT1 is low-affinity and noncompetitive, and only occurs at high concentrations of calcium [141]. On the other hand, increased intracellular calcium by iron overload was universally observed in various cellular models, as shown by direct iron treatment and hepcidin-induced iron uptake [143,144].

Like zinc, the potential transport mechanism of iron through calcium transporters has been reported, especially under iron-overloaded conditions such as non-transferrin-bound iron (NTBI) uptake pathways. LTCC has been shown to be highly activated in tissues with iron overload in various pathologic states [145]. In rat hearts and myocytes, LTCC showed a capacity to take up the reduced iron Fe^2+^ [146]. The Fe^2+^ influx through LTCC was validated with LTCC blocker in guinea pigs and mice, as well as in human osteoblast cells [146,147,148]. More recent studies have suggested that T-type calcium channels (TTCC) constitute another alternative iron transporter. In ventricular myocytes of thalassemic mice under iron overload, TTCC blocker prevented iron uptake to a much greater extent than other iron transporter blockers [149]. The Fe^2+^ entry pathway through TTCC was also validated in TTCC-expressed HEK293 cells [150].

With regard to RyR, Fe^2+^ seems to compete with Ca^2+^. RyR was shown to be inhibited by Fe^2+^, which in turn induces reduction in SR Ca^2+^ release in isolated rat cardiac SRs [151]. The effects of iron on RyR are controversial, as Fenton reaction-mediated ROS generation enhances RyR activity to release Ca^2+^ from ER in neuronal cells, which will be discussed in the section below [152,153]. In cardiac lysates from iron-deficient mice, RyR2 protein and mRNA levels were decreased, further indicating the relationship between iron and cardiac tissue, wherein levels too high or low are detrimental [154].

Ca^2+^ transport through iron transporters has also been suggested. Despite its central role in iron metabolism, ferroportin activity was shown to require Ca^2+^ as a cofactor for transport activity [155]. Furthermore, it was reported that ferroportin can transport Ca^2+^, with a single binding site for Ca^2+^ [156]. Transferrin receptor (TfR)-mediated iron transport was accelerated by Ca^2+^ through the activation of protein kinase C (PKC) [157]. More recently, the regulation of TfR by CaMKKII-CaMKIV signaling was proposed as CaMKIV knockout mice showed increased iron deposition in the cerebellum with abnormally high membrane-associated transferrin, suggesting the modulation of calcium signaling-mediated TfR trafficking [158,159,160].

#### 4.2.2. Iron and Calcium ROS Generation and Ferroptosis

The interaction between iron and other mineral ions, including calcium, is known to be linked to the production of reactive oxygen species (ROS) and the process of ferroptosis, which is attributed to the Fenton reaction [142,161]. Calcium, in particular, has been found to exacerbate iron-mediated ROS generation, especially in the context of neurodegenerative diseases [162,163,164,165]. In rat brain and neuronal cells, Fe^2+^-mediated lipid peroxisome rapidly induced increased Ca^2+^ uptake by cells, further leading to ROS generation [152,166].

As Ca^2+^ and Fe^2+^ both inhibit the expression of HIF-1α, a gene responsible for adapting hypoxia and oxidative stress, decreased HIF-1α was suggested as one mechanism for the synergistic effects of elevated Ca^2+^ and Fe^2+^ on ROS generation [162]. Also, ROS generation has been shown to increase IP3R, RyR, and TRPC channels, which are responsible for Ca^2+^ release, and iron regulatory protein 1, which induces more Fe^2+^ uptake. This further exacerbates lipid peroxidation, creating a vicious cycle of aggravated oxidative stress by calcium and iron [152,153,162,167,168]. Although it has not been mechanistically examined, Fe^2+^-induced oxidative stress, presumably, is involved in other redox-sensitive Ca^2+^-regulated enzymes such as CaMKII and calcineurin.

Given the interconnection between iron, ROS, and calcium, calcium has been proposed to play a role in ferroptosis. Although the exact role of calcium signaling in ferroptosis has not been mechanistically established, studies using ferroptosis inhibitors (Erastin) and inducers (RSL3) have revealed the relevance of calcium within this process [162,163]. For instance, the knockdown of Orai1, Orai3, and SOCE channels had a protective effect against ferroptosis [169]. Also, it was observed that ferroptosis can be mediated by the PKCα activation that is involved in calcium signaling, which activates MAPK/ERK signaling in neuronal cells. This suggests the potential involvement of calcium signaling in ferroptosis, not only through the sequential accumulation of ROS [170]. In addition, CaMKKII has been observed to suppress ferroptosis, via restraining lipid peroxidation, through the activation of Nrf2-dependent antioxidative machinery [171]. As the regulation of ROS and antioxidant levels plays a role in senescent cell progression, these iron-related mechanisms may play a role in the progression of senescence and various types of metal dyshomeostasis.

## 5. Conclusions

**Figure 2:**
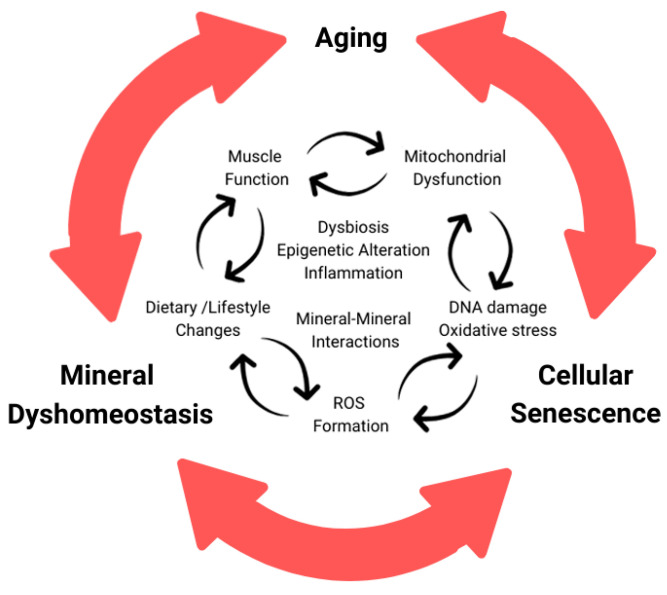
Cycle of dyshomeostasis in aging organism related with minerals and cellular senescence. Chronological aging causes accumulation of DNA damage, oxidative stress, and various alterations progressing cellular senescence. During cellular senescence and aging, increased ROS generation and disrupted mineral homeostasis, among other phenotypes, are seen. This altered mineral content could cause disruption between various minerals including, but not limited to calcium, iron, and zinc. These interactions are interconnected and can build off one another, causing a damaging cycle.

The essential micronutrient calcium serves many functions, and this review has focused on its role in muscle, aged muscle, and cellular senescence. We have observed various changes in regulatory mechanisms, some of which are altered while others remain constant. The review has also delved into the complex interactions between calcium and other minerals such as zinc and iron. Despite the focus on zinc and iron, it is critical to note that calcium has similarly intertwined relationships with many other metals, minerals, and compounds. Within cells, calcium regulation occurs in various organelles, including the ER/SR, mitochondria, and the nucleus, and these regulations can change with age, cell stress, or cellular senescence. Although many strides have been taken in recent years, there are still gaps in knowledge regarding the relationships with neurodegenerative diseases, specific muscles and their various fiber types, and novel mineral–mineral interactions. Some of these areas are currently under investigation, with ongoing human clinical trials of senolytics therapies and the adaptation of new methodologies such as improved calcium probes in mechanistic-focused studies. The dysregulation of mineral concentration in aging and cellular senescence may be driven by a cycle of dyshomeostasis. This cycle involves the underlying mechanisms of aging progressing towards inflammation and cellular senescence, leading to altered mineral concentrations and interactions. This further exacerbates the negative cycle, advancing aging and age-related diseases, not only in the muscle but also in various other tissues throughout the aging organism, as summarized in Figure 2.

## Figures and Tables

**Table 1:** Age-associated changes in muscle.

Author/Year	Species/Age	Finding	Implication	Ref.
**Human Studies**				
Goodpaster, B.H. et al. (2006)	Three-year changes in old (70–79 years; *n* = 1880) subjects (female, 51%; male, 48%)	Initially functioning older adults exhibited three-fold greater loss in strength than the loss of muscle mass over the course of 3 years. Maintenance or even gain of lean mass did not necessarily prevent loss of strength.	Loss of strength is more rapid and suggests a decline in the quality of muscle. Losses of strength can increase risks of falls and serious injury.	[33]
Venturelli, M. et al. (2014)	Young (25 ± 2 years; *n* = 12), old-mobile (87 ± 3 years of age; *n* = 12) and 12 old-immobile (88 ± 4 years; *n* = 12) sex-matched subjects (female, *n* = 9; male, *n* = 3)	Mean skeletal telomere length of thigh decreased with age, but not of arm. Mean free radical increased with age in thigh but not in arm.	Chronological age does not affect the cellular aging of skeletal muscle evenly. Physical inactivity could be mediated by the free radical effect.	[30]
Murgia, M. et al. (2017)	Young (22–27 years; *n* = 4) and old (65–75 years of age, *n* = 4) non-sarcopenic subjects	Fiber size of fast-twitch (type 2a) but not slow-twitch (type 1) muscles decreased with age. Decreased respiratory chain complexes were found in aging muscles. Changes in protein quality, turnover, and metabolic pathways were changed with age in muscles.	Many glycolytic enzymes were expressed higher in slow fibers of the older cohort, and these same enzymes declined within fast-twitch fibers, showing changes in mitochondria in line with previous studies. These proteomic data support the idea that aging may differentially affect type 2 muscle fibers, protein homeostasis, mitochondria function, and metabolic pathways.	[34]
Walton, R.G. et al. (2019)	Randomized, double-blind trials in placebo (*n* = 55) and Metformin (*n* = 54) groups of old (over 65 years) subjects	Metformin inhibited progressive resistance training-induced lean mass gain but did not change the effect of weight loss from training. Metformin prevented decreases in type 1 fiber frequency.	Metformin inhibits gains in fat-free mass in response to concurrent aerobic and resistance training in subjects with prediabetes. Metformin may inhibit hypertrophy via mTORC1 inhibition.	[35]
Therakomen, V. et al. (2020)	Old (over 60 years, *n* = 330) male subjects	Development of sarcopenia is positively correlated with age, as is prefrailty and low physical activity.	The study supports previously understood risk factors for primary sarcopenia: age, prefrailty, physical activity, and nutritional status, but not sex.	[36]
Hester, G.M. et al. (2021)	Young (*n* = 15, age = 20.7 ± 2.2 years) and old (*n* = 15, age = 71.6 ± 3.9 years) male non-sarcopenic subjects	Peak torque was lower in the older group at all velocities compared to the young group. The whole-muscle cross-sectional area was smaller in old muscles. Type 1 fiber was larger and type 2 fiber was smaller in muscles in the older group.	Microbiopsy methods appear to be viable alternatives that are less intrusive but with similar results. The lack of association among neuromuscular junction deterioration, strength, and age-related muscle fiber atrophy may be due to the fact that samples were from a non-sarcopenic healthy elderly population.	[37]
Bres, E. et al. (2023)	Sarcopenic (*n* = 30) and non-sarcopenic (*n* = 22) old (over 70 years) subjects	Serum fibroblast growth factor (FGF) 19 was correlated with muscle ultrasound parameters of pennatation angle and muscle fiber length. FGF19 levels were not correlated with age, BMI, nutritional parameters, or tissue mass.	The association of FGF19 and the pennetation angle implies that a high-FGF19 environment promotes both the development of fast-twitch muscles as well as a negative association with balance and lower extremity strength, suggesting the role of FGF19 in muscle function and architecture.	[38]
**Animal studies**				
Lang, F. et al. (2018)	Manual denervation model of sarcopenia in adult C57BL/6J mice	Skeletal muscle exhibits varied protein changes after denervation, with opposing protein changes between type 1 and type 2a muscle fibers of Soleus during muscle atrophy.	Using a manual denervation method to study muscle atrophy, at 7 days post-denervation, this group showed complexities of response between different muscle fibers and tissues.	[39]
Lukjanenko, L. et al. (2020)	Young (9–13 weeks) and aged (20–25 months) C57BL/6J mice	Aging impaired fibro/adipogenic progenitor (FAP) functions with a failure to support muscle stem cells. Transcriptome analysis relieved the downregulated WNT1 inducible signaling pathway protein 1 (WISP1) gene comparing aged to young activated FAPs.	Aging damages functions of FAPs and their ability to support myogenesis, the regenerative capacity. WISP1 is a FAP-derived factor controlling muscle stem cell expansion and differentiation, and age-induced loss of WISP occurs due to a lack of FAP population.	[40]
Xu, H. et al. (2021)	Whole body CuZnSOD KO mice and muscle-specific mitochondrial targeted catalase (mMCAT) transgenic mice with C57BL/6J background. accelerated sarcopenia model in female C57BL/6J mice (*n* = 12)	An altered oxygen consumption rate and peroxide generation in CuZnSOD KO mice is reversed through mMCAT expression. Significant muscle loss and function is observed in CuZnSOD KO mice. Muscle fiber composition and diameter in CuZnSOD KO mice were decreased through mMCAT.	In an accelerated sarcopenia model, mMCAT is sufficient to prevent the majority of muscle atrophy and weaknessin CuZnSOD KO mice. The absence of CuZnSOD leads to a reduction in force and disruption of neuromuscular junction with increased mitochondrial ROS. Mitochondrial scavenging capacity is important for the prevention of the loss of innervation, muscle atrophy, and weakness.	[41]
Kim, K.H. et al. (2022)	Young (5 weeks) and aged (25 month) C57BL/6J female mice	Fecal microbiota transplantation of young mice improved grip strength, muscle fiber thickness, and other fitness markers. Young donor transplantation increased genes involved in cell differentiation, proliferation, and fatty acid synthesis in muscle.	A group of Bacteroidetes from the young-derived microbiota are discriminative in old mice, increasing gene expression in recipients’ muscles and skin. This supports that the age related changes in the microbiome can play a role with changes in aging muscles.	[42]

## Data Availability

Not applicable.

## Abbreviations

Abbreviation	Full name
ATAD3A	ATPase family AAA domain containing 3A
CaM	calmodulin
CaMK	calmodulin kinase
CaMKK	calmodulin kinase kinase
CDKN	cyclin-dependent kinase inhibitor
CRD	cysteine-rich domain isoforms of the neuregulin-1
DHPR	dihydropyridine receptor
DMT	divalent metal transporter
ERK	extracellular signal-regulated kinase
FAP	fibro/adipogenic progenitor
FGF	fibroblast growth factor
GCV	ganciclovir
GLUT	glucose transporter type 4
HMGBI	high-mobility group box 1
HRC	histidine-rich Ca^2+^-binding protein
IGF1	insulin-growth like factor 1
IL-6	interleukin-6
IP3	inositol-1,4,5-triphosphate
IP3R	inositol-1,4,5-triphosphate receptor
IR	insulin receptor
IRS	insulin receptor substrate
ITPRKO	inositol 1,4,5-trisphosphate receptorknock out
KO	knock out
LETM1	leucine zipper-EF-hand-containing transmembrane protein 1
LTCC	L-type calcium channel
MAPK	mitogen-activated protein kinase
MCAT	mitochondrial targeted catalase
MCU	mitochondrial calcium uniporter
MFI	multidimensional fatigue inventory
MG	mitsugumin 29
mPTP	mitochondrial permeability transition pore
mTOR	mammalian target of rapamycin
NCS	neuronal calcium sensor
NF-κB	nuclear factor kappa B
NFAT	nuclear factor of activated T cells
NOX1	NADPH oxidase
Nrf2	nuclear factor-erythroid factor 2-related factor 2
PI3K	phosphoinositide 3-kinase
PIP3	phosphatidylinositol-3,4,5-triphosphate
PKC	protein kinase C
PLC	phospholipase C
PRDX3	Peroxiredoxin 3
ROS	reactive oxygen species
RyR	ryanodine receptor
SA-β-gal	senescence-associated beta-galactosidase
SASP	senescence-associated secretory phenotype
SERCA	sarcoplasmic/endoplasmic reticulum Ca^2+^-ATPase
SkM	skeletal muscle
SLC	solute carrier
SOCE	store-operated calcium entry
SOD	superoxide dismutase
SR	sarcoplasmic reticulum
STIM1	stromal Interaction Molecule 1
TAF	telomere-associated foci
TfR	transferrin receptor
TNF-a	tumor necrosis factor-alpha
TNiC	C-terminal one third of troponin I
TRPC	transient receptor potential canonical
TRPM	transient receptor potential melastin
TTCC	T-type calcium channels
VGCC	voltage-gated calcium channel
WT	wild type
ZIP	Zrt-/Irt-like protein
ZnT	zinc transporter
γ-H2AX	gamma-H2A histone family member X
